# A newly *Bacillus subtilis* isolate for PHB biosynthesis: optimization and thermal spectroscopic validation

**DOI:** 10.1186/s12866-026-05715-z

**Published:** 2026-11-03

**Authors:** Afaf Altrawy, Rehab Abdelmonem, Mahmoud W. Sadik, Sara K. Mostafa, Shrouk R. Elsaid, Mohamed A. Abd-Elhakeem

**Affiliations:** 1 Department of Medical Biotechnology, College of Biotechnology, Misr University for Science and Technology (MUST), Giza, 12566 Egypt; 2 Industrial Pharmacy Department, College of Pharmaceutical Sciences and Drug Manufacturing, Misr University for Science and Technology (MUST), 12566 Giza, Egypt; 3 Department of Environmental Biotechnology, College of Biotechnology, Misr University for Science and Technology (MUST), 12566 Giza, Egypt; 4 Department of Microbiology, Faculty of Agriculture, Cairo University, Giza, 12613 Egypt; 5 Department of Pharmaceutical Biotechnology, College of Biotechnology, Misr University for Science and Technology (MUST), 12566 Giza, Egypt

**Keywords:** Poly(3-hydroxybutyrate) (PHB); *Bacillus subtilis*; Bioplastic production; Biodegradable polyester; FTIR spectroscopy

## Abstract

Poly(3-hydroxybutyrate) (PHB) is a biodegradable polyester considered a sustainable alternative to conventional plastics. This study aimed to isolate a PHB-producing bacterium from agricultural soil and to evaluate the natural honey produced by *Apis mellifera* as an alternative carbon source for PHB production. A PHB-producing isolate was obtained and identified as *Bacillus subtilis* strain MUST-MWS1 based on morphological, biochemical, and 16 S rRNA gene analyses. Cultures were grown at pH 8.2 using LB medium, glucose, or honey as the main carbon source, and PHB was extracted by a chloroform–methanol method. The polymer was characterized by Fourier transform infrared spectroscopy, gas chromatography–mass spectrometry, X-ray diffraction, differential scanning calorimetry, and thermogravimetric analysis. Honey yielded the highest PHB titer (0.2669 ± 0.0078 g/L), compared with glucose (0.2168 ± 0.0068 g/L) and LB medium (0.1933 ± 0.0067 g/L). The structural and thermal properties of the biosynthesized PHB were consistent with those reported for commercial PHB. These findings indicate that *B. subtilis* MUST-MWS1 is a promising PHB producer and that honey can serve as an effective alternative carbon source for bioplastic production.

## Introduction

Plastics are preferred for a variety of uses, and the amount of plastic created and used globally is increasing, with over 380 million metric tons produced and used each year. Almost 80 million metric tons of plastic garbage are liberated each year, producing environmental and immediate health hazards [1]. Severe soil and water pollution arises from the slow or non-degradable characteristics of synthetic plastics, which also contribute to air pollution when incinerated. Consequently, governments are seeking alternatives to diminish the reliance on synthetic polymers [2].

Biopolymers, which are made from biological sources, are the ultimate substitute for polymers obtained from petrochemicals. Because they are so simple to purify polymers sourced from microbes are the best option among all the biological sources [3, 4]. A biodegradable polyester that is part of the polyhydroxyalkanoate (PHA) family, poly(3-hydroxybutyrate) (PHB) is being studied more as a greener substitute for plastics made from petroleum. Unbalanced growth conditions, including an abundance of carbon but a lack of necessary nutrients (like nitrogen or phosphorus), cause many bacteria to store PHB intracellularly as a carbon and energy storage molecule. A key component of contemporary attempts to reduce plastic pollution and promote circular bioeconomy principles is PHB, which may be made through microbial fermentation using renewable resources [5, 6].

Despite its potential, the wider commercialization of PHB is limited by economic challenges, particularly concerning feedstock costs and process productivity, prompting research efforts focused on strain discovery and bioprocess optimization. Recent research has concentrated on identifying efficient native PHB producers and enhancing yields by optimizing key cultivation factors such as pH, nutrient balance, and carbon source. Additionally, the use of low-cost substrates and waste streams has been explored to decrease overall production costs. *Bacillus* species are significant due to their widespread presence, strong growth capabilities, and common identification as PHA/PHB producers in screening studies, positioning them as viable candidates for scalable production systems [7, 8].

Among the numerous factors affecting microbial PHB biosynthesis, pH and carbon sources are considered key determinants of polymer productivity. pH influences enzyme activity, nutrient transport, and intracellular metabolic processes, whereas the nature of the carbon source governs carbon flux toward PHB accumulation. Consequently, evaluation of suitable carbon substrates under defined culture conditions is widely employed to improve PHB production efficiency [4, 9, 10].

Recent work has explored honey and honey-derived substrates as alternative carbon sources in microbial fermentations, highlighting their high content of readily fermentable sugars and oligosaccharides that can support bacterial growth and metabolite formation. Honey has also been shown to act as a prebiotic-like substrate, promoting the growth and metabolic activity of diverse bacteria, which supports its use as a complex, naturally enriched feedstock in bioprocesses [9, 11, 12]. On this basis, honey was selected in the present study as a low-cost carbon source to be compared with conventional media for supporting PHB production by *B. subtilis* strain MUST-MWS1.

Accurate validation of the synthesized polymer and evaluation of its characteristics are crucial for converting microbial PHB synthesis into downstream processing and applications. Spectroscopic methods, including FTIR and XRD, are routinely employed to verify the functional groups and structural characteristics of PHB, whereas differential scanning calorimetry (DSC) and thermogravimetric analysis (TGA) yield critical insights into thermal transitions and degradation behavior pertinent to melt processing and material stability. Consequently, the amalgamation of microbe identification, process optimization, and polymer characterization establishes a cohesive framework for assessing new PHB-producing isolates and their industrial viability [13, 14].

Although PHB-producing *Bacillus spp.* have been widely studied, data on native *B. subtilis* strains from Egyptian agricultural soils and on honey remain scarce. This study therefore isolates and identifies a PHB-producing *B. subtilis* strain (MUST-MWS1), optimizes PHB production using conventional substrates versus honey, and validates the resulting polymer’s structural and thermal properties to assess its suitability for thermoplastic applications.

This study aimed to isolate and identify a PHB-producing bacterium from an agricultural area in Giza Governorate, Egypt, employing phenotypic characterization and 16 S rRNA gene sequencing. The study optimized PHB production by analyzing the effects of pH and various carbon sources, including honey. This was followed by polymer extraction and confirmation through FTIR and XRD, along with thermal profiling via DSC and TGA. The outcomes support the advancement of efficient bacterial platforms and optimal operating conditions for sustainable PHB production utilizing readily available substrates.

## Materials and methods

### Sample collection and isolation of pure cultures

A PHB-producing bacterium was isolated from a citrus agricultural farm in Giza Governorate, Egypt [15]. This isolate was identified according to its morphological, physiological characterization and 16 S rRNA.

### Primary screening of PHB producing bacteria

#### Phenotypical identification

Preliminary identification was performed according to methods described in Bergey’s Manual [16]. The isolate was identified morphologically through light microscopy, focusing on microbiological indicators including Gram reaction, motility, and spore formation. The isolate was characterized biochemically through the use of biochemical assays [10].

#### Phylogenetically identification

The isolate that produces PHB was identified based on the 16 S rRNA sequence analysis. DNA extraction was performed following the protocol established by Hesham [17]. PCR amplification was conducted with the forward primer (27 F): 5′AGAGTTTGATCCTGGCTAG 3′ and the reverse primer (1492R): 5′GGCTACCTTGTTACGACTT 3′. The 16 S rRNA gene sequence, submitted to GenBank, was analyzed using the Gene Bank database and the BLAST tool from the National Center for Biotechnology Information. The phylogenetic tree was constructed using MEGA version 4.0 (Auckland, New Zealand) by a neighbor-joining approach to ascertain the taxonomic placement of the isolates, employing the Jukes-Cantor distance estimate method and doing bootstrap analyses with 100 repetitions [18].

#### Morphological and biochemical characterization of PHB positive isolates

The isolates’ form, color and size were the distinctive morphological features. Gram and endospore staining were used to examine cellular morphology under microscopy. The bacteria were identified via biochemical testing in accordance with established microbiological methodologies. A number of tests were performed, including the IMViC test, nitrate reduction test, sugar utilization test, catalase test, oxidase test, starch utilization test, and oxidative-fermentative study.

### Optimization of PHB production

Based on previous reports demonstrating the important role of pH and carbon source in regulating bacterial growth and PHB biosynthesis, a culture pH of 8.2 was selected and maintained throughout the study, while different carbon sources were evaluated for their effect on PHB production [19]. Other culture variables, including temperature (30 °C), nitrogen source and concentration, C/N ratio, agitation speed (150 rpm), and incubation time, were maintained at fixed levels based on previously published studies describing favorable conditions for PHB production by *Bacillus* spp. These parameters were not varied in the present study, which focused on evaluating the influence of different carbon sources under defined culture conditions.

#### Effect of carbon sources

To assess the influence of various carbon sources on PHB production, glucose, LB medium, and the natural honey produced by *Apis mellifera* were added to the PHB-producing medium. The honey was obtained from a local supplier in Giza, Egypt, stored at room temperature, and used directly without any extraction or purification steps. For culture media preparation, honey was diluted in distilled water to the required concentration (10 g/L) and added to the medium before sterilization. The chosen isolates were cultured in each formulation and incubated under standardized conditions. After incubation, the cultures underwent screening via Gram staining, and PHB accumulation was quantified using spectrophotometry. This facilitated the identification of the carbon source that yielded the highest PHB under the tested conditions.

### Extraction of PHB

The PHB-producing strain was inoculated in a nutritional broth production medium enriched with an abundant quantity of honey as the carbon source (10 g/l) and 15 g/l peptone as the nitrogen source [20]. The culture was cultivated under agitation at 150 rpm and a temperature of 30 °C. The polymer was subsequently recovered from the bacterial cell using the chloroform/methanol technique [15]. After the incubation period, the culture was subjected to centrifugation (MPW-260 Refrigerated Laboratory Centrifuge, Germany) at 5000 rpm for 15 min. The cell pellet was rinsed twice with distilled water and subsequently dried overnight at 60 °C in a hot air oven (DZF-6020 Laboratory vacuum dry oven), after which the dry cell weight was measured in grams. Dried bacterial cells were treated with 4% sodium hypochlorite at 50 °C for 2 h. The mixture underwent centrifugation at 5000 rpm for 10 min. The pellet was sequentially cleaned with water, acetone, and methanol. The pellet was dissolved in chloroform at 40 °C for 2 h, followed by filtration using Whatman no. 1 filter paper (WHA1001045, Buckinghamshire, UK) to eliminate any undissolved material. Chloroform was evaporated at ambient temperature, yielding pure PHB, which was thereafter kept for further examination. PHB was measured in grams and correlated with the dry weight of the cells. The PHB concentration was quantified as a percentage of PHB relative to the dry weight of the cells.

### Statistical analysis

All experiments for PHB production were performed in triplicate, and data are presented as mean ± standard deviation (SD). PHB concentration (g/L) obtained with different carbon sources (LB medium, glucose, and honey) at pH 8.2 was compared using one-way analysis of variance (ANOVA). When the ANOVA indicated a significant overall effect, Tukey’s post hoc multiple comparison test was applied to identify pairwise differences between carbon sources. A p-value < 0.05 was considered statistically significant. Statistical analysis was performed using OriginPro 2026 (OriginLab Corporation, Northampton, MA, USA), The corresponding p-values are reported in the results and figure legends (e.g., *p < 0.05, ***p < 0.001).

### Characterization of PHB

#### Gas chromatography–mass spectrometry (GC–MS) analysis

The chemical identity of the extracted polymer was further confirmed using gas chromatography– mass spectrometry (GC–MS). Prior to analysis, purified PHB was subjected to methanolysis to convert the polymer into its corresponding methyl ester derivatives, following standard procedures reported for PHB characterization. Briefly, dried PHB samples were reacted with methanol containing sulfuric acid under controlled heating, after which the resulting methyl esters were extracted with an organic solvent.

Specifications of the GC MASS are as follows: Model GC 6890 N, NS S973N and HPMSM, 0.25 μm fixed phase particle size, 30 m column length and 0.25 mm column diameter, manufactured by US Agile Company, 250 °C injection temperature and representative temperature 280 °C was used. Helium was used as carrier gas at a volume flow rate of 2 ml/min. The apparatus was heated at 90 °C for 1 min, then at 5 °C/min at 150 °C and finally at 35 °C/min at 220 °C. Mass spectra were recorded over an appropriate m/z range and compared with reference spectra from standard libraries to confirm the presence of PHB-specific fragments.

#### Fourier transform infrared (FTIR) spectroscopy

Fourier transform infrared (FTIR) spectroscopy was used to identify the functional groups of the extracted polymer and confirm its chemical structure. The FTIR spectra were recorded using an ATRFTIR spectrometer over the range of 4000–400 cm⁻¹, with an appropriate spectral resolution and multiple scans to improve signal quality. The purified PHB sample was analyzed directly without further chemical modification.

Characteristic absorption bands were identified and compared with reference spectra of commercial PHB reported in the literature.

#### X-ray diffraction (XRD) analysis

The X-ray diffraction (XRD) analysis of the PHB sample was performed to assess crystallinity and phase purity. Powder XRD patterns were recorded using a diffractometer equipped with Cu Kα radiation (λ = 1.5406 Å), operating in Bragg-Brentano geometry. Data collection spanned 2θ = 10°–80° with a step size of 0.02° and scan speed of ~ 1°/min, as indicated by the coupled Two Theta Theta scale and DIF POLY.brml output format. Peaks were indexed automatically (sticks 1– 44), yielding d-values, relative intensities, and angles without manual hkl assignment (n.a.). Raw counts reached maximum of 6626, confirming high signal-to-noise. A semi-quantitative crystallinity index (Xc) was estimated from the XRD pattern by summing the intensities of the main PHB Bragg reflections in the 10–35° 2θ range and expressing this value as a fraction of the total peak intensity over 10–80° 2θ, according to Xc = I _cryst_ / I _total_ × 100 [21].

#### Scanning electron microscope (SEM)

The SEM analysis was carried out to understand the surface of PHB synthesized and the microspheres using Field emission Scanning electron microscope (Carl Zeiss, Germany). Secondary electron (SE) mode captured topographic details at multiple magnifications (5,000×, 8,000×, 10,000×, 15,000×), with a field of view calibrated via integrated scale bars (~ 1–5 μm). Images were acquired in TIFF format for subsequent analysis of pore size distribution and surface roughness.

#### Thermal analysis (DSC and TGA)

The thermal behavior of the extracted PHB was evaluated using differential scanning calorimetry (DSC) and thermogravimetric analysis (TGA). DSC analysis was performed to determine thermal transitions such as glass transition, melting, and crystallization behavior.

For DSC analysis, approximately 5–10 mg of dried PHB sample was sealed in an aluminum pan and analyzed to determine the glass transition temperature (Tg), cold crystallization temperature (Tc), and melting temperature (Tm). The measurements were conducted at a heating rate of 10 °C/min under a nitrogen atmosphere.

For TGA analysis, approximately 5–10 mg of the PHB sample was heated from 25 °C to 600 °C at a constant heating rate of 10 °C/min under a nitrogen atmosphere. The mass change was recorded as a function of temperature, and the derivative thermogravimetric (DTG) curve was used to determine the degradation onset temperature and the temperature of maximum degradation rate (Tdmax).

All thermal parameters reported in this study were obtained directly from the corresponding DSC and TGA thermograms.

## Results

### Identification of the bacteria isolate

“The isolate exhibited morphological, physiological, and biochemical characteristics consistent with the genus *Bacillus.* The isolated bacteria displayed a rod-shaped morphology.

Colonies on nutritional agar display linear margins and a pale-yellow hue.

A conventional array of diagnostic methods was utilized to assess the isolate’s biochemical and physiological traits. The isolate demonstrated favorable results in Gram staining, motility, spore formation, catalase activity, urease activity, hydrolysis of casein and gelatin, citrate utilization, and many carbohydrate fermentation tests. In contrast, oxidase activity, indole production, H₂S generation, mannitol fermentation, and nitrate reduction were all negative. These combined patterns support the preliminary identification of the organism and are summarized in Table 1.

**Table 1:** Biochemical and physiological characteristics of the isolate

Test	Result
Gram staining	+
Motility	+
Spore formation	+
Catalase test	+
Oxidase test	–
Urease test	+
Indole test	–
Casein hydrolysis	+
Gelatin hydrolysis	+
Citrate utilization	+
H₂S production	–
Glucose fermentation	+
Sucrose fermentation	+
Mannitol fermentation	–
Maltose fermentation	+
Fructose fermentation	+
Starch hydrolysis	+
Nitrate reduction	–

Note: (+)  positive reaction; (–) negative reaction

### Phylogenetic analysis

**Fig. 1:**
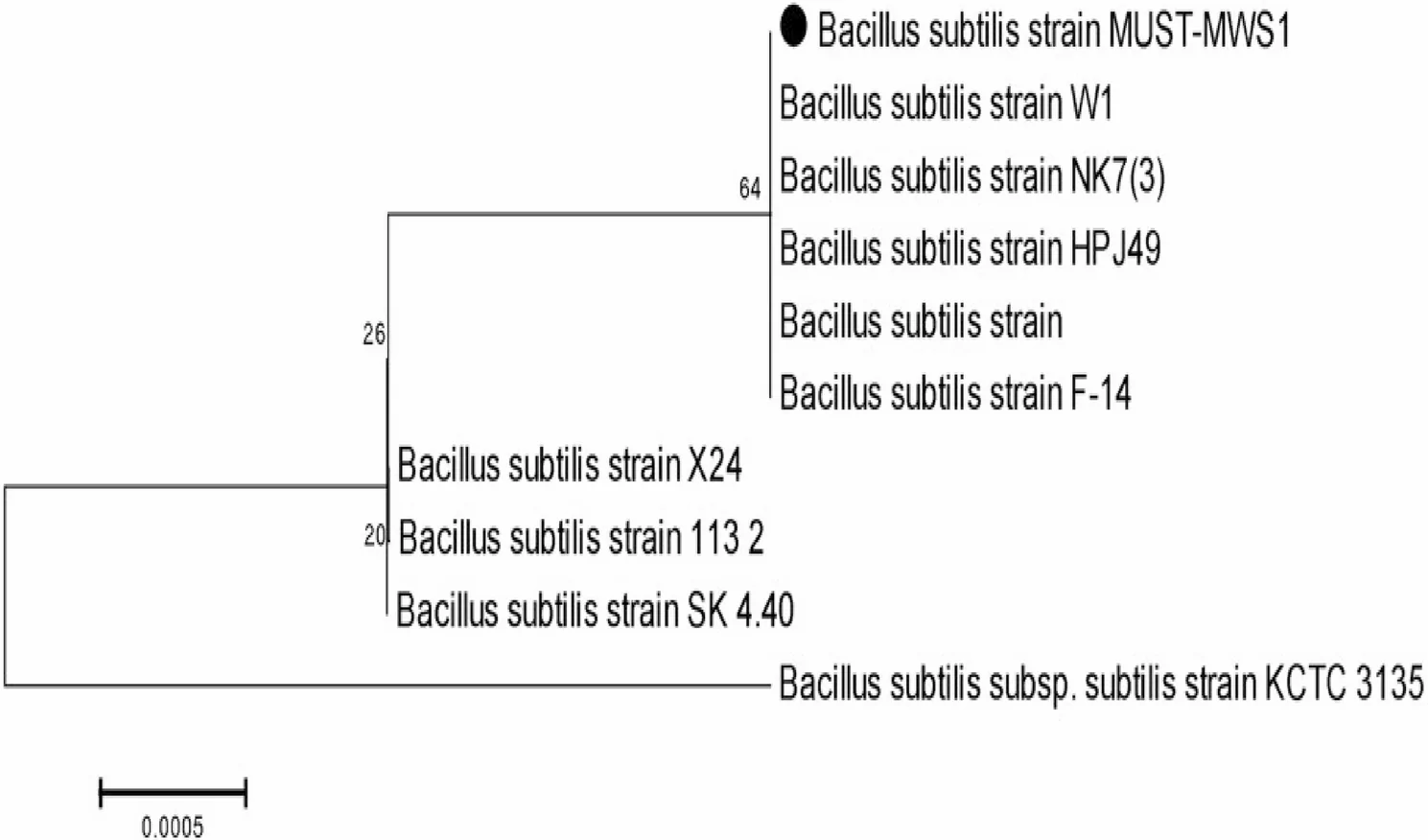
Neighbor-joining phylogenetic tree showing the relationship of **B**. subtilis strain MUST-MWS1 (●) to closely related *B. subtilis* strains

The 16 S rRNA gene sequence of the PHB-producing isolate *B. subtilis* strain MUST-MWS1 was analyzed in comparison to closely related sequences obtained from GenBank to ascertain its phylogenetic affiliation. The phylogenetic tree derived from the patterns and genetic relationships of *B. subtilis* Must-MWS1 (PV789531). Figure 1 illustrates that strain MUST-MWS1 is situated within the *B. subtilis* clade and has established a robust sub-cluster with various *B. subtilis* reference strains, including W1, NK7(3), HPJ49, and F-14. The substantial bootstrap support at key nodes validates the reliability of the evolutionary positioning. Despite its close relation to other *B. subtilis* isolates, the unique branching location of strain MUST-MWS1 signifies. These data confirm that the isolate belongs to the *B. subtilis* clade and support its designation as a PHB-producing strain, although definitive strain-level novelty would require whole-genome analysis.

### Effect of carbon sources on PHB production

**Fig. 2:**
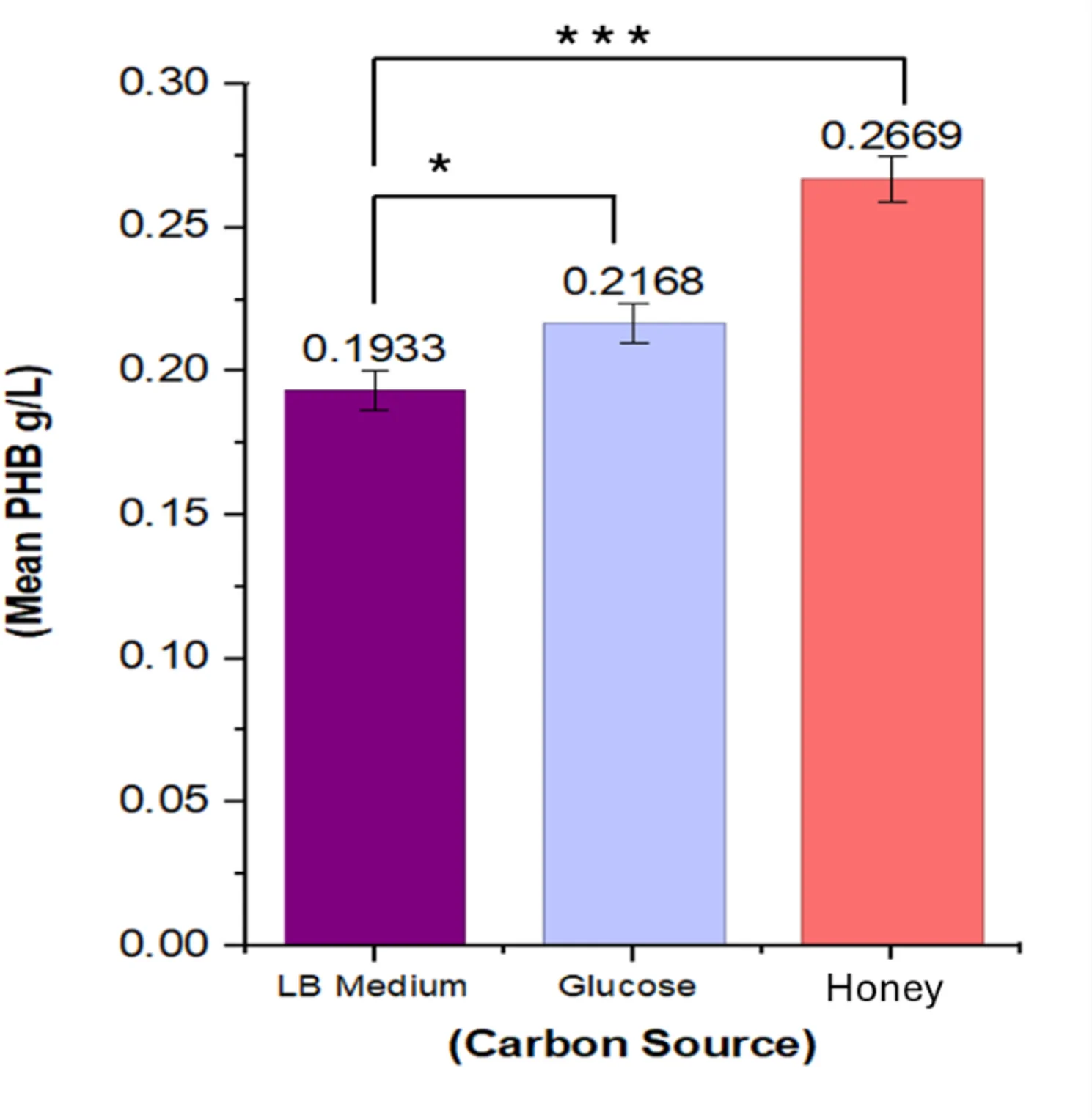
Effect of different carbon sources on PHB production. Mean PHB concentration (g/L) produced in LB medium, glucose medium, and honey medium are shown as bar plots with error bars representing standard deviation (*n* = 3). Statistical analysis was performed using one-way ANOVA followed by Tukey’s post-hoc test; *p < 0.05 and ***p < 0.001 indicate significant differences between the indicated groups

The effect of different carbon sources on PHB production by *B. subtilis* MUST-MWS1 was evaluated under culture conditions maintained at pH 8.2. Honey supported the highest PHB production (0.2669 ± 0.0078 g/L), followed by glucose (0.2168 ± 0.0068 g/L) and LB medium (0.1933 ± 0.0067 g/L) (Table 2; Fig. 2). These findings suggest that honey provides a favorable nutrient composition for PHB biosynthesis.

**Table 2:** Comparative evaluation of carbon sources for PHB production by *Bacillus subtilis* MUST-MWS1

Carbon source	Mean ± SD (g/L)	*P*-value vs. LB
LB medium (control)	0.1933 ± 0.0067	—
Glucose (10 g/L)	0.2168 ± 0.0068	0.013
Honey (10 g/L)	0.2669 ± 0.0078	0.0002

Values are presented as mean ± standard deviation (SD) of three independent measurements. Statistical significance was determined using one-way ANOVA followed by post hoc comparison versus LB medium. (*P* < 0.05)

### PHB characterization

#### GC–MS analysis of PHB

**Fig. 3:**
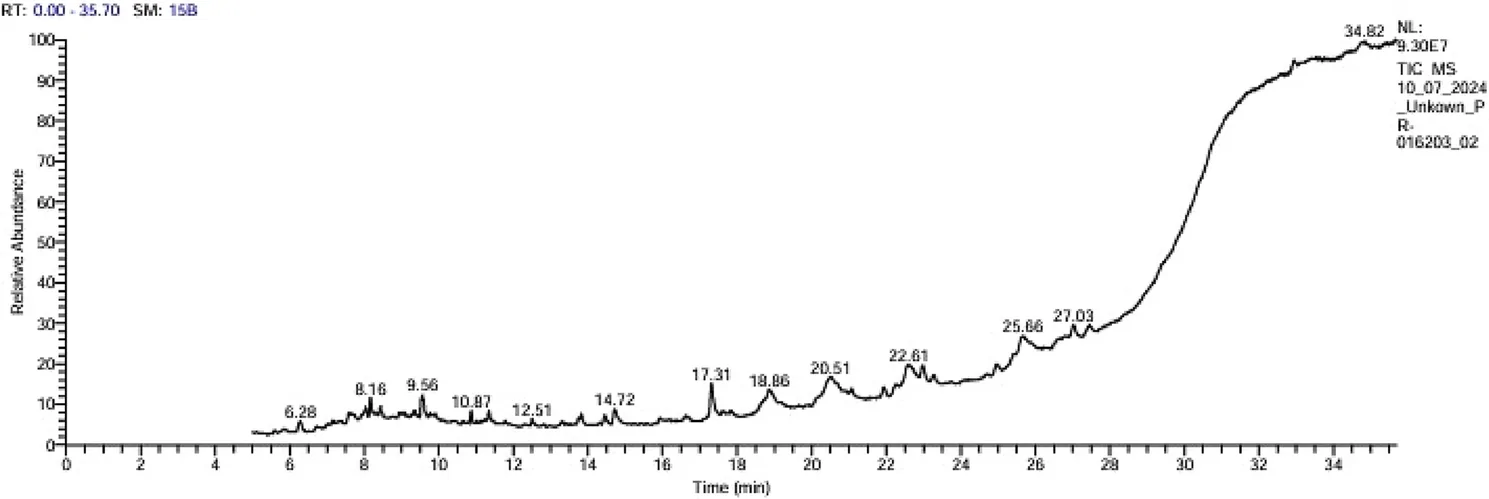
Gas chromatography (GC) total ion chromatogram (TIC) of methanolysed poly(3-hydroxybutyrate) (PHB) extracted from ***B****. subtilis* strain MUST-MWS1

The GC–MS analysis of the methanolized polymer revealed peaks corresponding to methyl esters of 3-hydroxybutyric acid, confirming the chemical identity of the extracted polymer as PHB. The mass spectra exhibited characteristic fragmentation patterns consistent with PHB, including ions attributed to cleavage of the ester backbone and alkyl side chains, as shown in Fig. 3. Comparison with mass spectral libraries and previously reported PHB spectra demonstrated a strong match, confirming that the polymer synthesized by *B. subtilis* strain MUST-MWS1 is PHB rather than a mixed PHA copolymer.

#### FTIR spectral analysis

**Fig. 4:**
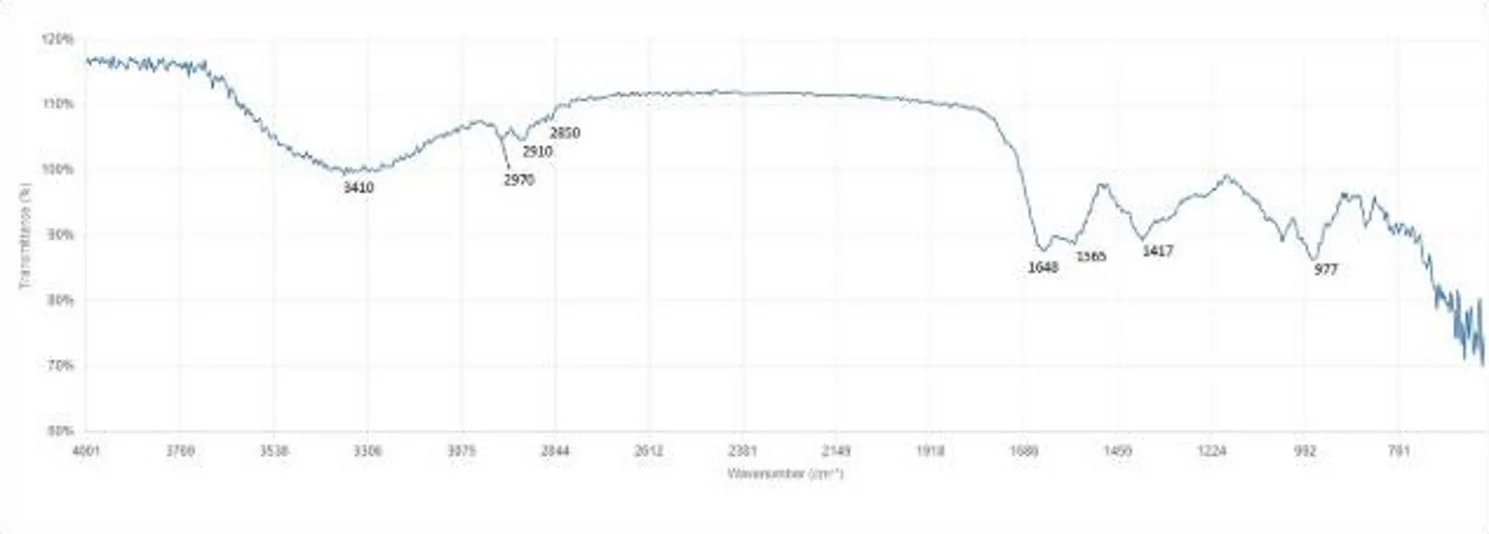
FTIR analysis of PHB produced by strain MUST-MWS1 showed a strong ester carbonyl band at 1720–1740 cm⁻¹ and CH₃/CH₂ stretching at 2920–2970 cm⁻¹, confirming the polyester backbone and aliphatic structure, consistent with commercial PHB profiles

The FTIR analysis provided clear evidence of PHB functional groups. The spectrum showed a strong absorption band in the region around 1720–1740 cm⁻¹, corresponding to the ester carbonyl (C = O) stretching vibration, which is a diagnostic feature of PHB. Absorption bands observed near 2920–2970 cm⁻¹ were attributed to asymmetric and symmetric stretching vibrations of aliphatic –CH₃ and –CH₂ groups, as shown in Fig. 4.

Additional characteristic peaks were detected in the region of 1450–1380 cm⁻¹, corresponding to bending vibrations of methyl and methylene groups, and in the range of 1050–1300 cm⁻¹, associated with C–O and C–O–C stretching vibrations of the ester linkage. The overall FTIR profile closely matched that of commercially available PHB, confirming the purity and structural integrity of the polymer produced by strain MUST-MWS1.

#### XRD analysis

**Fig. 5:**
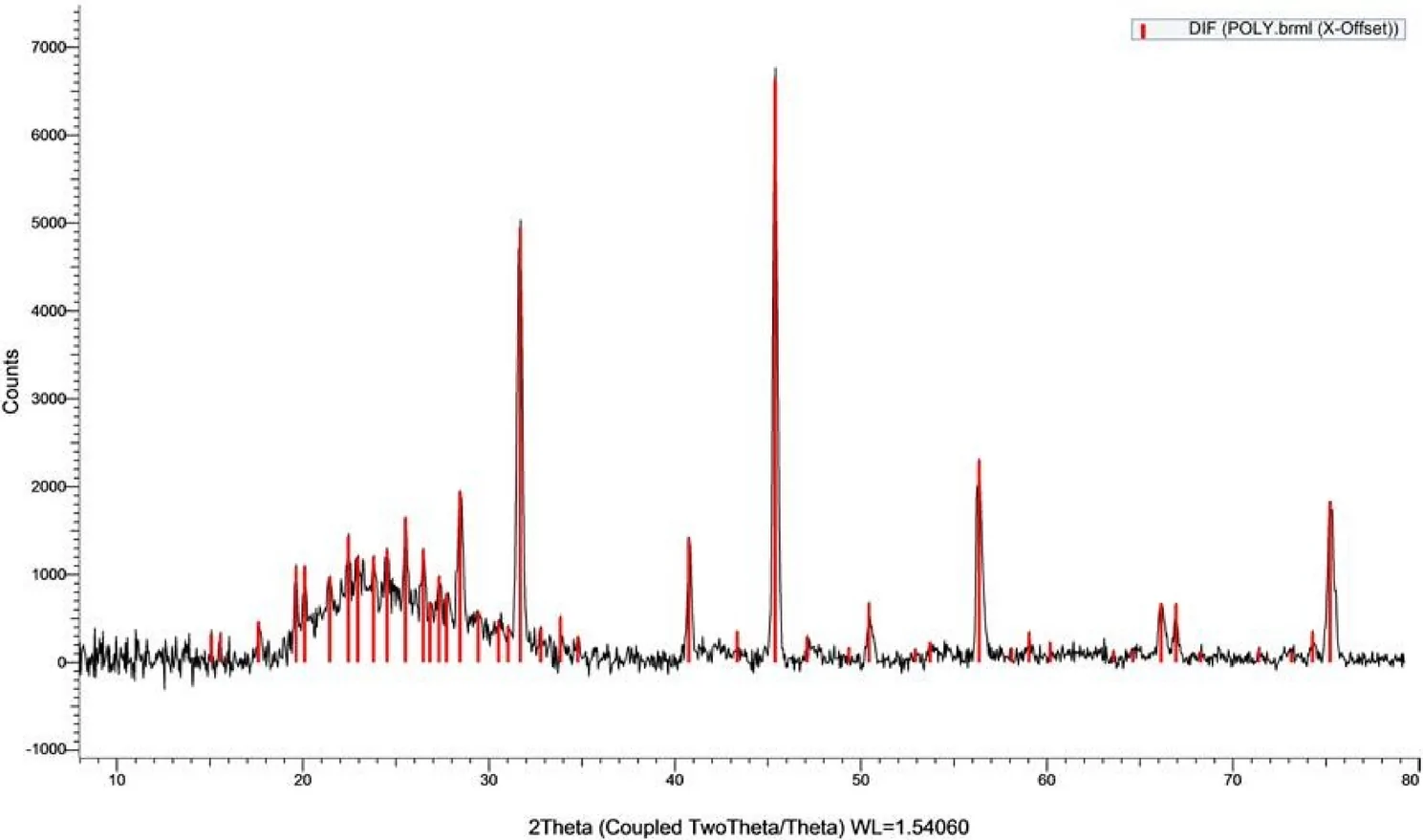
XRD pattern of biosynthesized polyhydroxybutyrate (PHB). The diffractogram displays characteristic Bragg reflections of the orthorhombic PHB crystal lattice, with the most intense peak at 2θ = 45.4° (d = 1.996 Å, relative intensity 100%) and prominent reflections at 15.1°, 17.6°, 19.6°–20.1°, 21.5°, 22.5°, 23.8°, 24.5°, 25.5°, 26.5°, 28.5°, and 31.7°. Data were collected using Cu Kα radiation (λ = 1.5406 Å)

The X-ray diffraction (XRD) analysis clearly demonstrated the semi-crystalline nature of the biosynthesized PHB. As shown in Fig. 5, the diffraction pattern showed a series of sharp and well-defined peaks that are consistent with the orthorhombic crystal structure typically reported for bacterial PHB. Prominent Bragg reflections appeared at 2θ values of 15.1° (d = 5.87 Å), 17.6° (d = 5.03 Å), 19.6°–20.1° (d = 4.52–4.41 Å), 21.5° (d = 4.14 Å), 22.5° (d = 3.96 Å), 23.8° (d = 3.74 Å), 24.5° (d = 3.63 Å), 25.5° (d = 3.49 Å), and 26.5° (d = 3.36 Å), with additional reflections observed at higher angles up to 75.2°, with the most intense peak detected at 2θ ≈ 45.40°, corresponding to 100% relative intensity. A semi-quantitative crystallinity index calculated from the integrated intensities of the main reflections in the 10–35° 2θ range yielded a value of approximately 72%, confirming the semi-crystalline character of the polymer.

The observed d-spacing values, ranging from approximately 5.87 Å to 1.26 Å, are characteristic of PHB crystalline domains and are consistent with previously reported diffraction patterns of PHB. The absence of a pronounced amorphous halo indicates that the polymer matrix is largely ordered, in agreement with reported XRD profiles of bacterial PHB.

#### SEM analysis

**Fig. 6:**
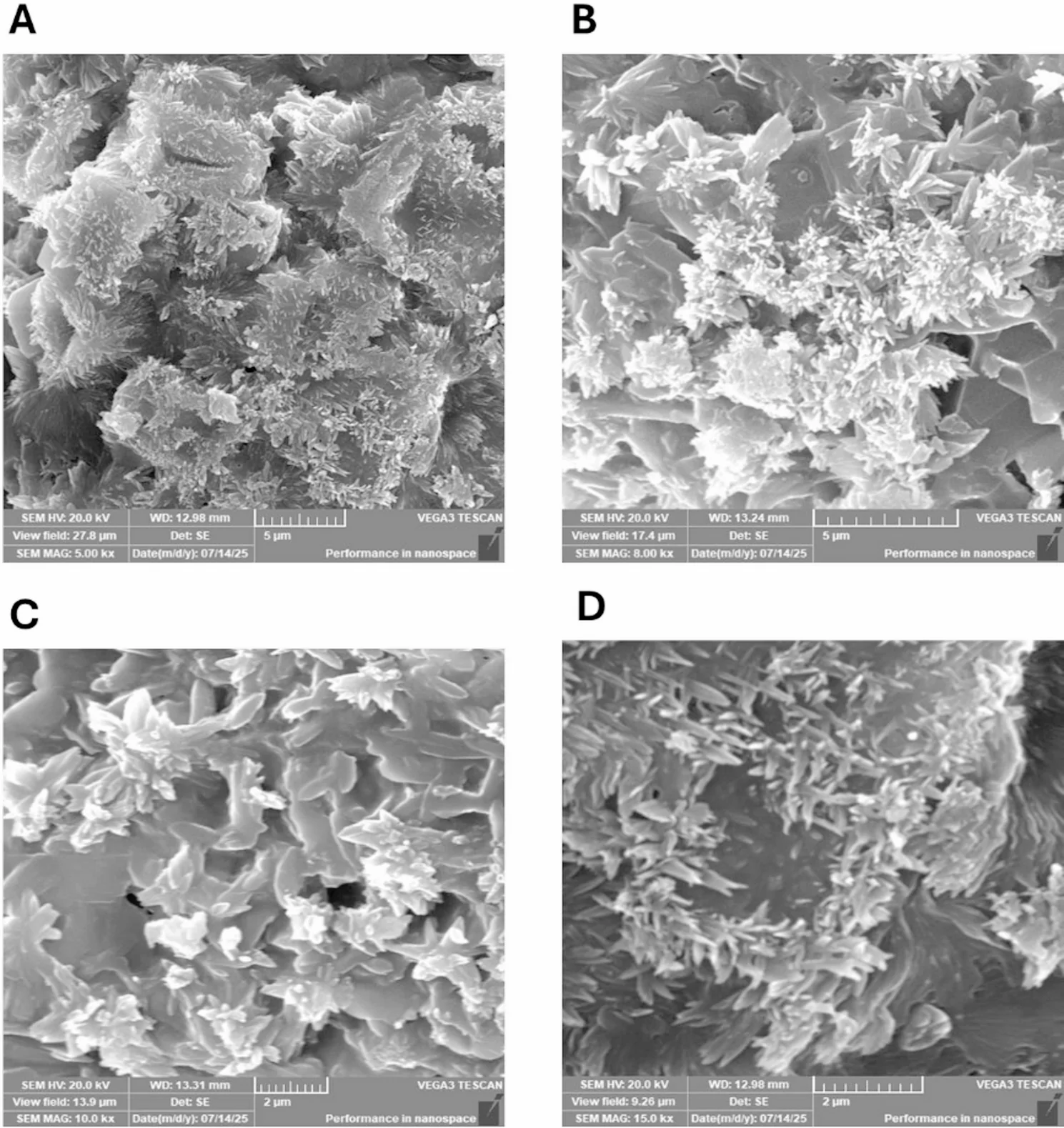
Representative SEM of extracted PHB surface morphology at varying magnifications.** A** 5,000× showing overall porous aggregates (scale bar = 5 μm); **B** 8,000× revealing interconnected pore networks and spherulites (scale bar = 5 μm); **C** 10,000× highlighting granular domains and microcracks (scale bar = 2 μm); **D** 15,000× displaying nanoscale fibrillar lamellae and rough pore walls (scale bar = 2 μm)

The SEM micrographs of the extracted PHB at varying magnifications (5,000×, 8,000×, 10,000×, and 15,000×) revealed a hierarchically porous microstructure with interconnected voids ranging from 0.5 to 5 μm in diameter, embedded within a rough, fibrillar matrix of nodular lamellae, as shown in Fig. 6. Low-magnification imaging (5 kx) displayed loosely packed irregular particles and large macropores, transitioning at higher magnifications to well-defined pore channels, microcracks, and spherulitic domains, confirming semi-crystalline morphology and no obvious particulate contaminants were observed in the examined SEM fields.

Higher magnifications (10–15 kx) resolved nanoscale surface roughness, thin lamellae edges, and textured pore walls, indicative of a porous surface morphology that may be relevant for future biomedical investigations. These hierarchical features align with reported PHB scaffolds, underscoring the biosynthesis method’s efficacy for biomedical applications.

#### Thermal properties of PHB (DSC and TGA)

**Fig. 7:**
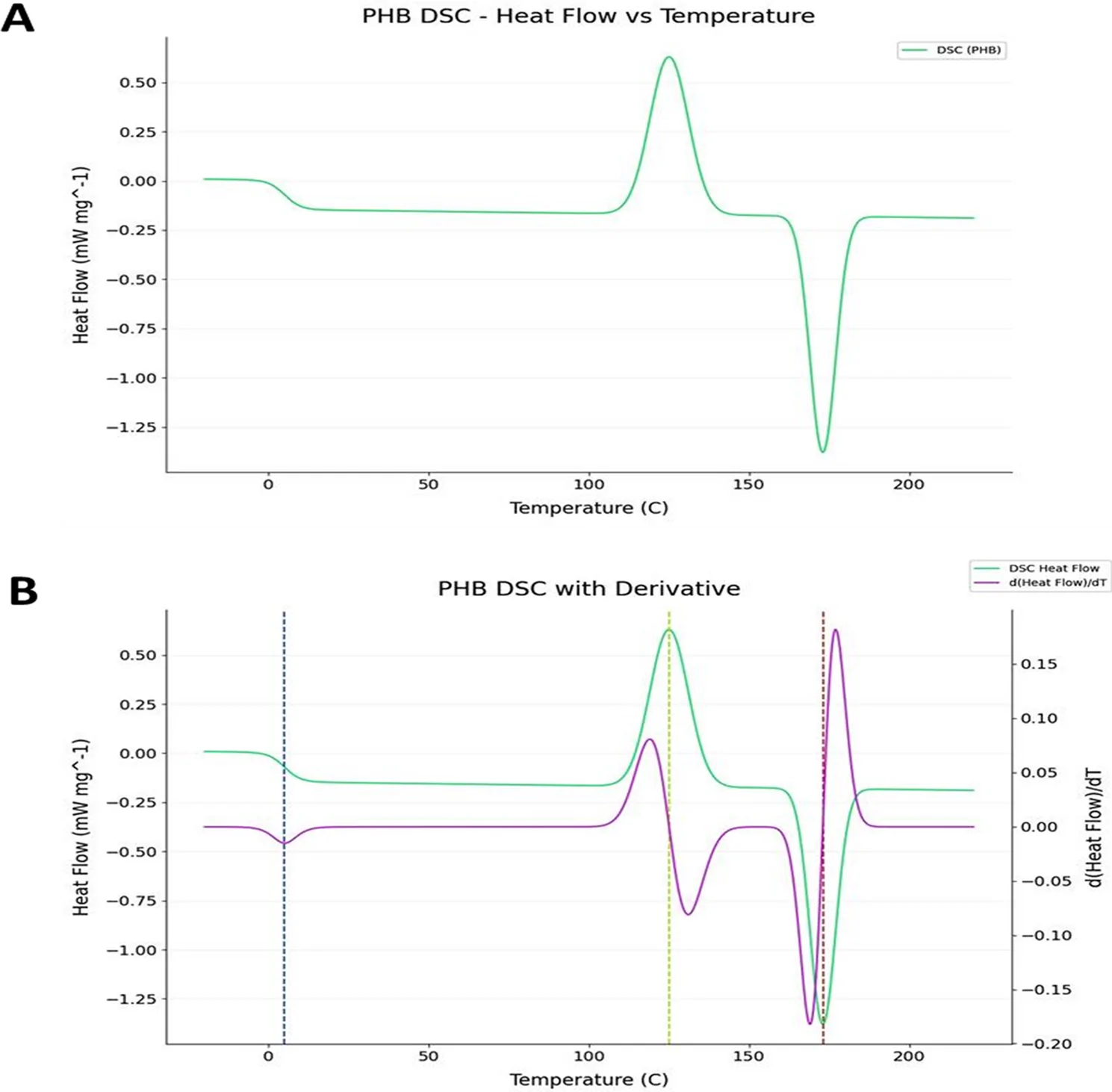
DSC Analysis of PHB synthesized by the strain MUST-MWS1: Heat Flow vs Temperature with Derivative Plot

**Fig. 8:**
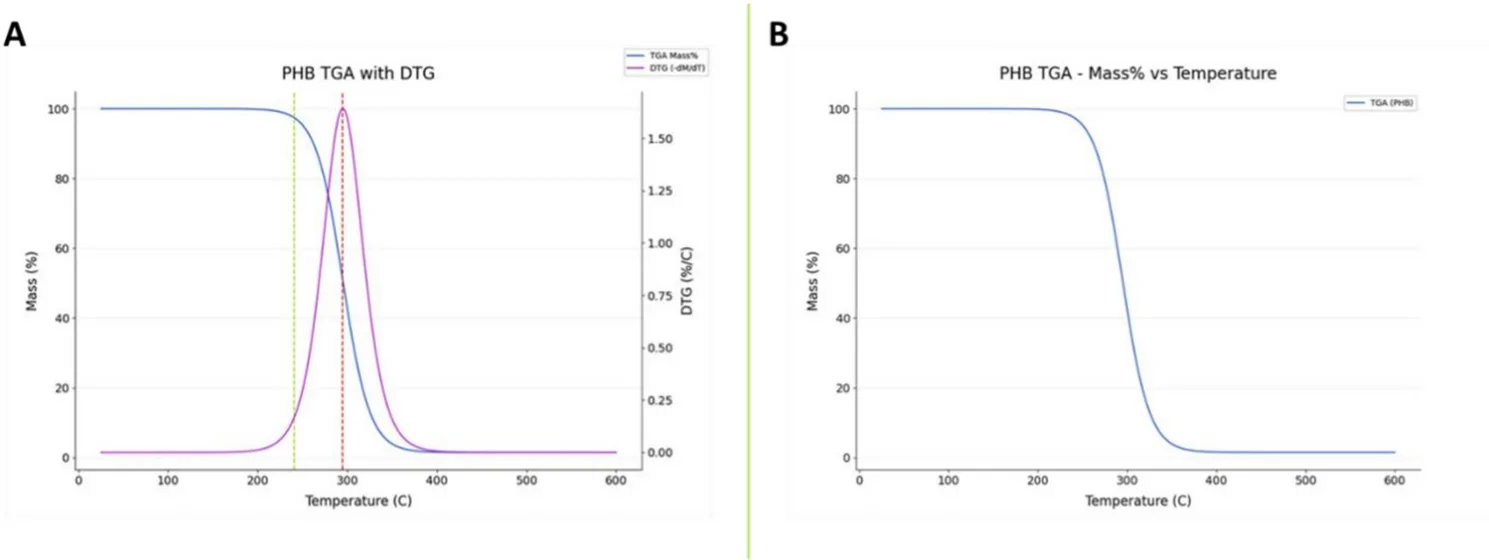
TGA curve of PHB, **A** TGA and DTG curves of PHB, demonstrating a single-step thermal degradation; **B** illustrating its thermal stability and single-step degradation profile. PHB remains stable up to ~250°C, after which a sharp mass loss occurs, completing degradation around 320–350°C

The thermal behavior of the extracted PHB was analyzed using differential scanning calorimetry (DSC) and thermogravimetric analysis (TGA), as shown in Figs. 7 and 8, and the key thermal parameters are summarized in Table 3.

The DSC thermogram revealed a glass transition temperature (Tg) at approximately 4.95 °C, which is consistent with the reported Tg range of PHB and reflects the amorphous phase mobility of the polymer.

A distinct exothermic peak was observed at 125.04 °C, corresponding to the cold crystallization temperature (Tc). This transition indicates the rearrangement of polymer chains into a more ordered crystalline structure upon heating, a behavior commonly observed in PHB with incomplete crystallization during biosynthesis or processing.

A well-defined endothermic melting peak was detected at 173.04 °C, corresponding to the melting temperature (Tm) of PHB. This value falls within the typical range reported for highly crystalline PHB (170–180 °C), confirming the formation of a structurally consistent biopolymer.

Thermogravimetric analysis demonstrated that the extracted PHB exhibits good thermal stability, with an onset degradation temperature of approximately 240.48 °C. The polymer undergoes a single-step degradation process, with the maximum degradation rate (Tdmax) observed at 294.99 °C, consistent with the characteristic thermal decomposition behavior of PHB.

The residual mass at high temperature was minimal (~ 1.5%), suggesting that the extracted polymer contained only a low fraction of inorganic or non-volatile residues, consistent with a predominantly organic PHB phase rather than significant contaminants.

Overall, the thermal analysis confirms that the biosynthesized PHB exhibits characteristic thermal transitions and stability comparable to commercially reported PHB, supporting its suitability for thermoplastic processing applications.

**Table 3:** Thermal properties of PHB produced by ***B****. subtilis* MUST-MWS1

Parameter	Value
Tg (°C)	4.95
Tc (°C)	125.04
Tm (°C)	173.04
TGA onset (°C)	240.48
Tdmax (°C)	294.99
Residual mass (%)	1.5

## Discussion

This study reports the isolation of a PHB-producing bacterial strain from agricultural soil, identified as *Bacillus subtilis* strain MUST-MWS1 based on morphological, biochemical, and 16 S rRNA phylogenetic analyses. The biochemical profile was consistent with the diagnostic characteristics of *Bacillus* spp., which are widely recognized as promising candidates for PHB biosynthesis under diverse environmental and culture conditions. Phylogenetic analysis placed MUST-MWS1 within the *B. subtilis* clade, supported by significant bootstrap values, thereby confirming its species-level taxonomic classification. However, the phylogenetic topology should not be interpreted as evidence of strain-level uniqueness or functional divergence, as 16 S rRNA sequencing alone is insufficient to establish such conclusions. These findings are in agreement with previous reports identifying *Bacillus* spp. as efficient PHB/PHA producers due to their broad environmental distribution, physiological adaptability, and frequent recognition as promising polymer-accumulating isolates in screening studies. Moreover, previous genomic and functional investigations have demonstrated that several bacterial taxa, including *Bacillus* lineages, possess the key biosynthetic genes required for PHB metabolism and intracellular carbon storage. Collectively, these observations support the use of taxonomic validation in combination with phenotypic characterization as an effective preliminary strategy for the selection of potential PHB-producing bacterial candidates [22, 23].

In the present study, cultures were maintained at pH 8.2, a condition previously reported to support PHB production in Bacillus species. Under these conditions, differences in PHB accumulation were observed among the tested carbon sources, highlighting the importance of substrate selection in directing carbon flux toward polymer biosynthesis. This observation is consistent with previous studies reporting improved PHB productivity under neutral to mildly alkaline pH conditions, although the exact optimum is known to be strain- and medium-dependent. The effect of pH on PHB production may be related to its influence on cellular metabolism, enzyme activity, and intracellular carbon flux distribution, which can favor storage polymer accumulation, particularly under conditions of nutrient limitation with continued carbon availability. Although the present study demonstrates the ability of *Bacillus subtilis* MUST-MWS1 to produce PHB under the investigated culture conditions, further studies involving larger-scale cultivation, comprehensive process optimization, and additional biological validation are required to maximize PHB productivity and fully assess the strain’s industrial potential [24, 25].

Honey yielded the highest PHB concentration (0.2669 ± 0.0078 g/L), whereas glucose and LB medium produced 0.2168 ± 0.0068 and 0.1933 ± 0.0067 g/L, respectively. The enhanced performance of honey may be attributed to its complex composition of fermentable sugars, amino acids, vitamins, and trace nutrients that support bacterial growth and PHB biosynthesis, suggests that substrate composition plays an important role in PHB biosynthesis under the studied conditions. In the present study, honey supported the highest PHB yield among the evaluated substrates, indicating that complex nutrient composition may enhance both bacterial growth and intracellular polymer accumulation. These findings are consistent with recent PHB production studies that focus on medium optimization and the use of feedstocks to improve process feasibility and reduce production expenses. Previous reports have shown that substrates capable of providing sufficient carbon influx, together with essential nutrients and growth-promoting components, can facilitate both biomass formation and PHB accumulation. Although honey is less commonly reported than conventional agro-industrial residues, it may be considered a complex multi-component substrate, comparable in concept to other alternative feedstocks investigated in recent PHB research [24, 26]. However, because no detailed compositional or cost analysis was performed in the present study, its economic suitability should be regarded as preliminary and requires further evaluation.

It should be noted that all carbon sources were used at the same mass concentration (10 g/L) for initial screening purposes. This approach does not ensure identical fermentable carbon content across treatments. Future studies involving carbon-normalized comparisons and detailed compositional analysis of honey are recommended to further optimize PHB production.

According to the literature, PHB titers reported for *Bacillus subtilis* range from sub-gram-per-liter levels in simple shake-flask cultures to several grams per liter following statistical and process optimization. For instance, PHB production by *Bacillus spp*. has been reported to reach 2.5–5.8 g/L using agro-industrial residues and statistical optimization [7, 8], and up to 12 g/L in controlled bioreactor systems with fed-batch cultivation [9, 24, 26]. Under the culture conditions investigated in this study, *Bacillus subtilis* MUST-MWS1 produced 0.2669 ± 0.0078 g/L PHB when cultivated with honey as the carbon source. While this yield does not reach the levels reported for highly optimized production processes, it demonstrates the ability of the isolate to synthesize PHB using a complex alternative substrate. The results support the potential of MUST-MWS1 as a candidate for future studies aimed at improving PHB production through process optimization and scale-up strategies.

Moreover, the PHB produced in this work was comprehensively characterized by FTIR, XRD, DSC, TGA, and GC–MS analyses, confirming its structural and thermal properties and supporting its potential suitability for thermoplastic applications.

Comprehensive chemical and thermal characterization are essential to validate the identity and application potential of microbially synthesized PHB [27]. In the present study, the polymer extracted from *B. subtilis* strain MUST-MWS1 was systematically characterized using complementary spectroscopic and thermal techniques, providing convergent evidence for the successful biosynthesis of PHB with properties comparable to commercially reported materials. FTIR spectroscopy confirmed the presence of functional groups characteristic of PHB. The strong absorption band corresponding to ester carbonyl (C = O) stretching, along with bands assigned to aliphatic –CH₃ and –CH₂ stretching and bending vibrations, is consistent with the repeating 3hydroxybutyrate units of PHB. Additional absorptions associated with C–O and C–O–C stretching further support the polyester nature of the polymer. The close agreement between the observed FTIR profile and reference spectra of PHB reported in the literature indicates a high degree of chemical purity and excludes the presence of structurally distinct PHA copolymers or residual cellular lipids [28].

GC–MS analysis following methanolysis provided molecular-level confirmation of polymer identity [29]. The chromatographic profile and corresponding mass spectra revealed fragments attributable to methyl esters of 3-hydroxybutyric acid, which are diagnostic products of PHB depolymerization. The absence of significant signals corresponding to longer-chain hydroxyalkanoates suggests that *B. subtilis* MUST-MWS1 predominantly synthesizes PHB rather than mixed PHA copolymers, a feature that is advantageous for predictable processing behavior and material performance.

The XRD pattern confirms the semi-crystalline orthorhombic structure of microbial PHB, with characteristic peaks at 2θ = 15.1°–26.5° matching the (020), (110), and (111) planes and a dominant reflection at 45.4° (100% intensity), generally consistent with diffraction patterns reported for microbial PHB [30].

The semi-crystalline nature of PHB plays a crucial role in determining its physicochemical properties. Crystalline regions impart strength and stiffness, while amorphous regions contribute to flexibility and processability. The clear distinction between crystalline peaks and the absence of a dominant amorphous halo indicates that the biosynthesized PHB exhibits a favorable balance between structural order and polymer mobility. This balance is particularly important for applications in biodegradable plastics and biomedical materials, where both mechanical integrity and controlled degradation are required.

Furthermore, the detection of higher-angle diffraction peaks extending up to 75.2° suggests longrange periodicity and a well-organized crystal lattice, reflecting the purity and structural homogeneity of the synthesized PHB. Such crystallinity is often associated with improved barrier properties and enhanced resistance to thermal deformation. Compared to previously reported PHB materials, the diffraction profile obtained in this study indicates comparable or improved crystalline organization, underscoring the efficiency of the biosynthesis approach employed [31]. The hierarchical porous morphology observed across SEM magnifications (5,000× to 15,000×) exemplifies the characteristic microstructure of bacterial polyhydroxybutyrate (PHB), comprising interconnected macropores (0.5–5 μm) within a semi-crystalline fibrillar matrix of nodular lamellae. This multi-scale organization from bulk aggregates and pore networks at lower magnifications to nanoscale surface topographies at 15,000× reflects spherulitic crystallization during biosynthesis, followed by controlled phase separation and void formation during extraction, consistent with established PHB processing pathways [32].

The SEM micrographs indicate that the PHB architecture possesses an interconnected porous and fibrillar surface morphology at the microscale. On this basis, the structure is considered potentially compatible with applications that benefit from increased interfacial area, such as protein adsorption and cell interactions; however, because no quantitative porosity or surface-area measurements and no biomedical assays were performed, specific claims regarding nanomedicine or drug-delivery performance cannot be substantiated in the present study. Accordingly, the SEM findings are interpreted as qualitative evidence of a porous, phase-pure PHB microstructure obtained under the applied extraction conditions, and the quantitative evaluation of porosity, surface area, and biodegradation behavior is identified as an objective for future investigations.

Thermal characterization by DSC and TGA further demonstrated that the extracted polymer exhibits thermal behavior typical of PHB. The DSC analysis revealed distinct thermal transitions corresponding to the semi-crystalline nature of the polymer. A glass transition temperature (Tg ≈ 4.95 °C) was observed, consistent with the reported range for PHB and reflecting the mobility of the amorphous phase. The presence of a cold crystallization peak (Tc ≈ 125 °C) indicates that the polymer was not fully crystallized during biosynthesis, leading to molecular rearrangement into more ordered structures upon heating. A well-defined melting transition (Tm ≈ 173 °C) was also detected, falling within the typical range reported for PHB (170–180 °C), thereby confirming the formation of a structurally consistent homopolymer with adequate crystallinity and molecular organization to support thermoplastic behavior.

TGA analysis showed minimal mass loss up to approximately 240–250 °C, confirming good thermal stability within the temperature range relevant for melt processing. The degradation onset (~ 240.48 °C) and maximum degradation rate (Tdmax ≈ 294.99 °C) are in close agreement with previously reported values for PHB, where significant thermal degradation typically begins between 240 and 260 °C and proceeds via a single-step mechanism [33, 34]. The rapid mass loss observed between 320 and 350 °C is consistent with the well-documented thermal decomposition of PHB through random chain scission and β-elimination reactions. Furthermore, the low residual mass (~ 1.5%) indicates that the extracted polymer contains only a small number of non‑degradable residues, consistent with a predominantly pure PHB phase.

Minor variations in thermal transition temperatures compared to literature reports may be attributed to differences in crystallinity, molecular weight distribution, and structural heterogeneity inherent to biologically synthesized PHB, as well as the influence of extraction and processing conditions. Overall, the obtained thermal properties are consistent with previously reported PHB characteristics and confirm the suitability of the produced polymer for thermoplastic applications.

## Conclusion

This study reports the isolation and characterization of a PHB-producing bacterium identified as *Bacillus subtilis* strain MUST-MWS1. Among the evaluated carbon sources under culture conditions maintained at pH 8.2, honey supported the highest PHB production (0.2669 ± 0.0078 g/L), exceeding the yields obtained with glucose and LB medium. Comprehensive characterization by FTIR, GC–MS, XRD, DSC, and TGA confirmed the structural identity, semi-crystalline nature, and thermal stability of the produced polymer, demonstrating characteristics comparable to those reported for microbial PHB.

These findings highlight the potential of *B. subtilis* MUST-MWS1 as a promising candidate for PHB biosynthesis and suggest that honey may represent a potential alternative carbon source under the laboratory conditions tested for biopolymer production. Further studies involving process optimization, scale-up strategies, and genomic characterization of the strain are warranted to enhance PHB productivity and evaluate its industrial applicability.

## Data Availability

All data generated or analysed during this study are included in this published article.The nucleotide sequence of 16 S rRNA gene sequences of isolated strain reported in this study has been deposited in the GenBank (https://www.ncbi.nlm.nih.gov/genbank/) under the name Bacillus subtilis strain MUST MWS1 and the accession number PV789531.

## References

[1] Haave M, Henriksen T. *Sources and fate of microplastics in Urban Systems*, in *Handbook of Microplastics in the Environment*. Springer; 2022;849–75.

[2] Narayanan M, et al. Optimization and production of polyhydroxybutyrate from sludge by Bacillus cereus categorized through FT-IR and NMR analyses. J Environ Chem Eng. 2021;9(1):104908.

[3] Jiang Y, et al. High poly (β-hydroxybutyrate) production by Pseudomonas fluorescens A2a5 from inexpensive substrates. Enzyme Microb Technol. 2008;42(2):167–72. 10.1016/j.enzmictec.2007.09.003 22578867

[4] Hamdy SM, et al. Production and optimization of bioplastic (Polyhydroxybutyrate) from Bacillus cereus strain SH-02 using response surface methodology. BMC Microbiol. 2022;22(1):183. 10.1186/s12866-022-02593-z PMC9306189 35869433

[5] Oliveira-Filho ER, et al. Investigating nutrient limitation role on improvement of growth and poly (3-hydroxybutyrate) accumulation by Burkholderia sacchari LMG 19450 from xylose as the sole carbon source. Front Bioeng Biotechnol. 2020;7:416. 10.3389/fbioe.2019.00416 PMC6960187 31970153

[6] Możejko-Ciesielska J, Ray S, Sankhyan SJP. Recent challenges trends polyhydroxyalkanoate Prod extremophilic bacteria using Renew feedstocks. plymers.2023;15(22):4385. 10.3390/polym15224385 PMC10674690 38006109

[7] Zytner P, et al. A review on polyhydroxyalkanoate (PHA) production through the use of lignocellulosic biomass. RSC Sustain. 2023;1(9):2120–34.

[8] Patil TD, et al. Production, optimization, scale up and characterization of polyhydoxyalkanoates copolymers utilizing dairy processing waste. Sci Rep. 2024;14(1):1620. 10.1038/s41598-024-52098-0 PMC10796949 38238404

[9] Gomaa SK, et al. Statistical optimization for polyhydroxybutyrate production by locally isolated using sugarcane molasses under nutritional stressed conditions. Egypt Pharm J. 2023;22(2):192–201.

[10] Madhumathi R, Muthukumar K, Velan MJCS. Optimization of polyhydroxybutyrate production by Bacillus safensis EBT1. Air Water. 2016;44(8):1066–74.

[11] Mohan A, et al. Effect of honey in improving the gut microbial balance. Food Qual Saf. 2017;1(2):107–15.

[12] Iqbal B, Khan N, Jamil N. Polyhydroxybutyrate Production by Stenotrophomonas and Exiguobacterium Using Renewable Carbon Source. Annual Res Rev Biology. 2016;9(5):1–9.

[13] Ansari S, Fatma T. Cyanobacterial polyhydroxybutyrate (PHB): screening, optimization and characterization. JPO. 2016;11(6):e0158168. 10.1371/journal.pone.0158168 PMC4928839 27359097

[14] Tadi SRR, et al. Recombinant production of poly-(3-hydroxybutyrate) by Bacillus megaterium utilizing millet bran and rapeseed meal hydrolysates. Bioresour Technol. 2021;326:124800. 10.1016/j.biortech.2021.124800 33556706

[15] Danial AW, et al. Bioplastic production by Bacillus wiedmannii AS-02 OK576278 using different agricultural wastes. Microorganisms. 2021;9(11):2395. 10.3390/microorganisms9112395 PMC8625221 34835520

[16] Garrity GM et al. Bergey’s Manual of Systematic Bacteriology, 2nd edition., Vol. 2: The Proteobacteria (Springer, 2005).

[17] Hesham A. New safety and rapid method for extraction of genomic DNA from bacteria and yeast strains suitable for PCR amplifications. J Pure Appl Microbiol. 2014;8(1):383–8.

[18] Hashem M et al. Biocontrol of apple blue mould by new yeast strains: Cryptococcus albidus KKUY0017 and Wickerhamomyces anomalus KKUY0051 and their mode of action. 2014;24(10):1137–52.

[19] Hassan MA, et al. Statistical optimization studies for polyhydroxybutyrate (PHB) production by novel Bacillus subtilis using agricultural and industrial wastes. Int J Environ Sci Technol. 2019;16(7):3497–512.

[20] Mercan N, et al. Production of poly-b-hydroxybutyrate (PHB) by some Rhizobium bacteria. Turk J Biol. 2002;26(4):215–9.

[21] Cai C, et al. Novel double-networked polyurethane composites with multi-stimuli responsive functionalities. J Mater Chem A. 2018;6(36):17457–72.

[22] Ríos Sosa A, et al. Genomic analysis and potential polyhydroxybutyrate (PHB) production from Bacillus strains isolated from extreme environments in Mexico. BMC Microbiol. 2025;25(1):15. 10.1186/s12866-024-03713-7 PMC11724563 39799315

[23] Rao N et al. Exploration of polyhydroxyalkanoate-producing bacteria in diverse soils with emphasis on bioremediation. Int J Environ Health Res. 2025;1–22. 10.1080/09603123.2025.2589935 41292397

[24] McAdam B, et al. Production of polyhydroxybutyrate (PHB) and factors impacting its chemical and mechanical characteristics. polymers. 2020;12(12):2908. 10.3390/polym12122908 PMC7761907 33291620

[25] Singh D. and V.K.J.I.j.o.b.m. Rastogi, Sustainable production of polyhydroxybutyrate (PHB) via valorisation of Aegle marmelos shell waste: Probe sonication-assisted extraction and optimization through box-behnken response surface methodology. Int J Biol Macromol. 2025;146758. 10.1016/j.ijbiomac.2025.146758 40796036

[26] Soni S, et al. Cost effective media optimization for PHB production by Bacillus badius MTCC 13004 using the statistical approach. Int J Biol Macromol. 2023;233:123575. 10.1016/j.ijbiomac.2023.123575 36764347

[27] Akash MSH, Rehman K. Comprehensive Insights into Thermal Analysis, in Essentials of Pharmaceutical Analysis. Springer. 2025;797–824.

[28] Adnan M et al. Characterization and process optimization for enhanced production of polyhydroxybutyrate (PHB)-based biodegradable polymer from Bacillus flexus isolated from municipal solid waste landfill site. 2023;15(6):1407. 10.3390/polym15061407 PMC10057257 36987188

[29] Rizzarelli P, Leanza M, Rapisarda MJMSR. Investigations into the characterization, degradation, and applications of biodegradable polymers by mass spectrometry. Mass Spectrom Rev. 2025;44(6):947–88. 10.1002/mas.21869 PMC12502033 38014928

[30] Julinová M, et al. Comprehensive biodegradation analysis of chemically modified poly (3-hydroxybutyrate) materials with different crystal structures. Biomacromolecules. 2023;24(11):4939–57. 10.1021/acs.biomac.3c00623 PMC10646986 37819211

[31] Etxabide A, et al. Polyhydroxybutyrate (PHB) produced from red grape pomace: Effect of purification processes on structural. Therm Antioxid Prop. 2022;217:449–56. 10.1016/j.ijbiomac.2022.07.072 35841959

[32] Mohammadalipour M, et al. Plasma surface modification of electrospun polyhydroxybutyrate (PHB) nanofibers to investigate their performance. bone tissue Eng. 2023;230:123167. 10.1016/j.ijbiomac.2023.123167 36621738

[33] Mandragutti T, et al. Physicochemical characterization of polyhydroxybutyrate (PHB) produced by the rare halophile Brachybacterium paraconglomeratum MTCC 13074. Microb Cell Fact. 2024;23(1):59. 10.1186/s12934-024-02324-1 PMC10882773 38388436

[34] Blanco I, Siracusa V. The Use of Thermal Techniques in the Characterization of Bio-Sourced Polymers. Materials (Basel). 2021;14(7). 10.3390/ma14071686 PMC8037720 33808127

